# [Retracted] MicroRNA‑379 suppresses cell proliferation, migration and invasion in nasopharyngeal carcinoma by targeting tumor protein D52

**DOI:** 10.3892/etm.2024.12577

**Published:** 2024-05-20

**Authors:** Xiaojun Zhao, Jiusheng Chu

Exp Ther Med 16:1232–1240, 2018; DOI: 10.3892/etm.2018.6302

Following the publication of this paper, it was drawn to the Editor’s attention by a concerned reader that certain of the Transwell cell invasion assay data shown in Fig. 3B on p. 1236 were strikingly similar to data that had already been published in different form in another article written by different authors at a different research institute [Sheng N, Tan G, You W, Chen H, Gong J, Chen, D, Zhang H and Wang Z: MiR-145 inhibits human colorectal cancer cell migration and invasion via PAK4-dependent pathway. Cancer Med 6: 1331-1340, 2017].

Owing to the fact that the contentious data in the above article had already been published prior to its submission to *Experimental and Therapeutic Medicine,* the Editor has decided that this paper should be retracted from the Journal. The authors were asked for an explanation to account for these concerns, but the Editorial Office did not receive a reply. The Editor apologizes to the readership for any inconvenience caused.

